# New players in end-protection: LIM-domain proteins associate with the shelterin complex

**DOI:** 10.18632/aging.100177

**Published:** 2010-07-16

**Authors:** Eros Lazzerini Denchi

**Affiliations:** Department of Genetics, The Scripps Research Institute, 10550 North Torrey Pines Road, La Jolla, CA 92037, USA

One of the vital activities performed by telomeres is the protection of chromosome ends. This allows cells to distinguish the tip of linear chromosomes from sites of DNA damage, and to prevent end-to-end chromosome fusions. The protective function of telomeres is lost when telomeres become critically short; a condition that is frequently found in aging cells [1]. In mammals end-protection is achieved by the localization of a six-protein complex, termed shelterin [2], to the terminal telomeric DNA. Of the six members that constitute this complex three bind directly to DNA: TRF1, TRF2 and POT1 while the others; TIN2, TPP1 and RAP1 are recruited to chromosome ends through protein-protein interactions [3]. Depletion of individual components of this complex is sufficient to render telomeres dysfunctional resulting in the activation of a DNA damage response [4-8]. Interestingly, components of this complex show a high degree of specialization being able to suppress different branches of the DNA damage response machinery. TRF2 is primarily involved in suppressing ATM kinase activity at chromosome ends [9], while TRF1 protects telomeres during replication suppressing the activation of a ATR-mediated DNA damage response [8]. POT1 on the other hand suppresses ATR activation throughout the cell cycle [9]. The major question, which for the most part remains to be addressed, is how can these proteins suppress the DNA damage response? A key step towards decoding this puzzle is the identification of additional proteins involved in end-protection. It seems likely that the shelterin complex does not act alone but needs to recruit accessory proteins in order to protect chromosome ends. Indeed, one such accessory protein was identified in the nuclease Apollo that is recruited to telomeres by TRF2 [10] and plays a critical role in end protection [11, 12]. In this issue of Aging Sheppard and Loayza report on a new family of protein that associate with the shelterin complex and might play a critical role in end-protection [13]. TRIP6, a member of the Zyxin family of proteins was identified as a POT1 interacting protein in a yeast two-hybrid screen. TRIP6 binds to several members of the shelterin complex including TRF2 and TIN2. Intriguingly, TRIP6 is not the only member of this family of proteins to interact with the shelterin complex since Sheppard and Loayza also report that an other member of the Zyxin family, called LPP, interacts with the same shelterin components. Using knockdown experiments Sheppard and Loayza report that inhibition of either TRIP6 or LPP result in loss of end-protection with the accumulation of DNA damage proteins at telomeres. This body of work suggests that members of the Zyxin family of proteins associate transiently with telomeres and play a role in end-protection. Additional work is required to establish what is the precise role of these proteins at chromosome ends. However, given the role of this family of proteins in other cellular processes, it is likely these proteins at telomeres serve as recruitment platforms for enzymatic activities. This would parallel the role of the Zyxin family member Ajuba in transcriptional repression. Ajuba is able to recruit to RAREs arginine methylase activity that in turns is essential to achieve transcriptional repression. In a similar manner TRIP6 and LPP might recruit enzymatic activities required to suppress the DNA damage response at chromosome ends. As such, Zyxin family members would bring at chromosome ends key tools required to disguise the tips of chromosomes end and to hide them from the DNA damage machinery. Future work will define what are the relative contribu-tion and the mechanism of action of this new family of proteins involved in end-protection.
